# Supplementary figures and images for: PTEN differentially regulates endocytosis, migration, and proliferation in the enteric protozoan parasite Entamoeba histolytica
Source: PLoS Pathog. 2022 May 2;18(5):e1010147. doi: 10.1371/journal.ppat.1010147 (PMC9122207; doi:10.1371/journal.ppat.1010147)

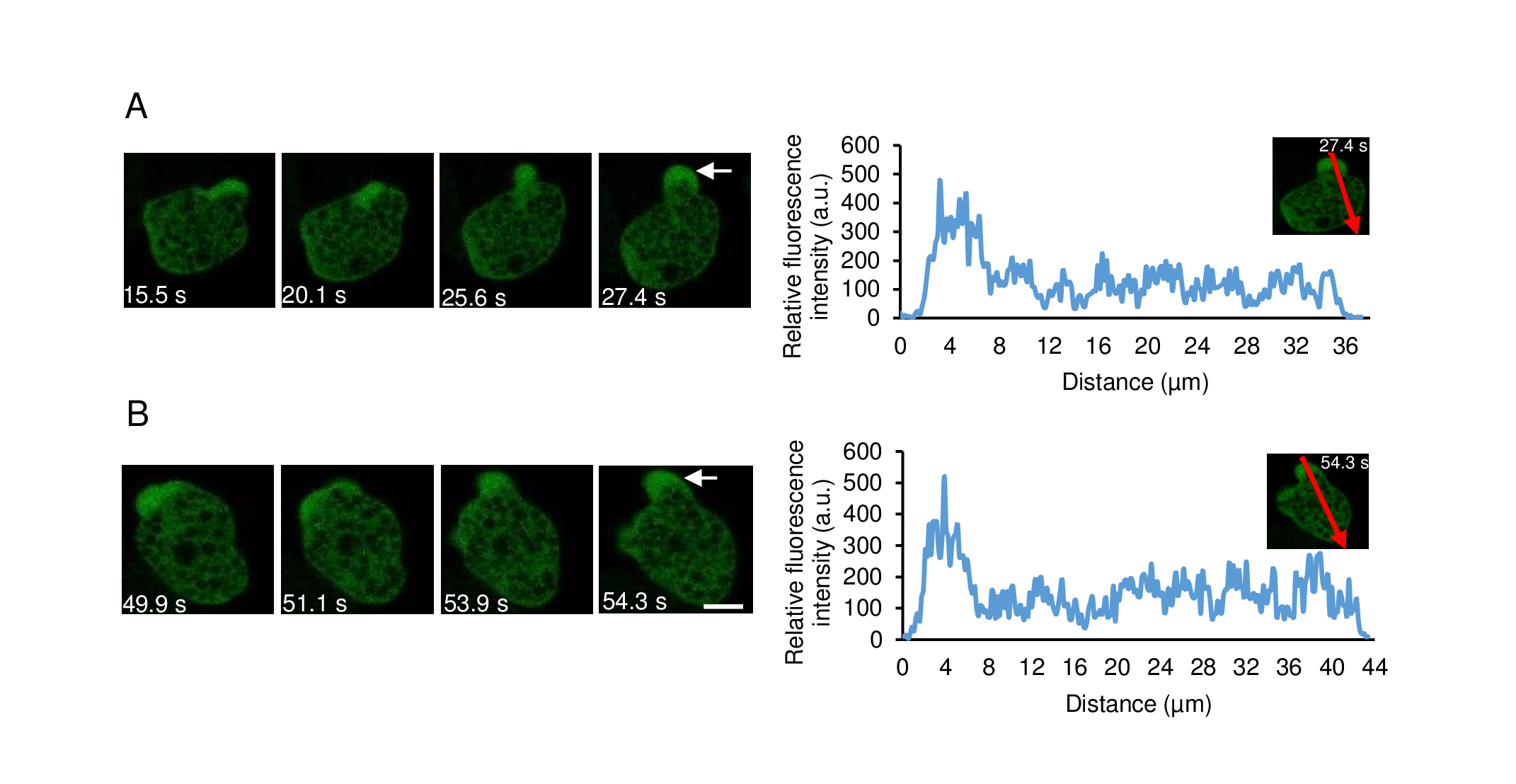

Supplement: S1 Fig — (A-B) Montage showing a time series of motile trophozoites expressing GFP-EhPTEN1 in left panels. The pseudopodal localization of GFP-EhPTEN1 is indicated by white arrow. The right panels show the fluorescence intensity of GFP-EhPTEN1 across the amoebic trophozoites. (Scale bar, 10 μm). (TIF) [file ppat.1010147.s001.tif]

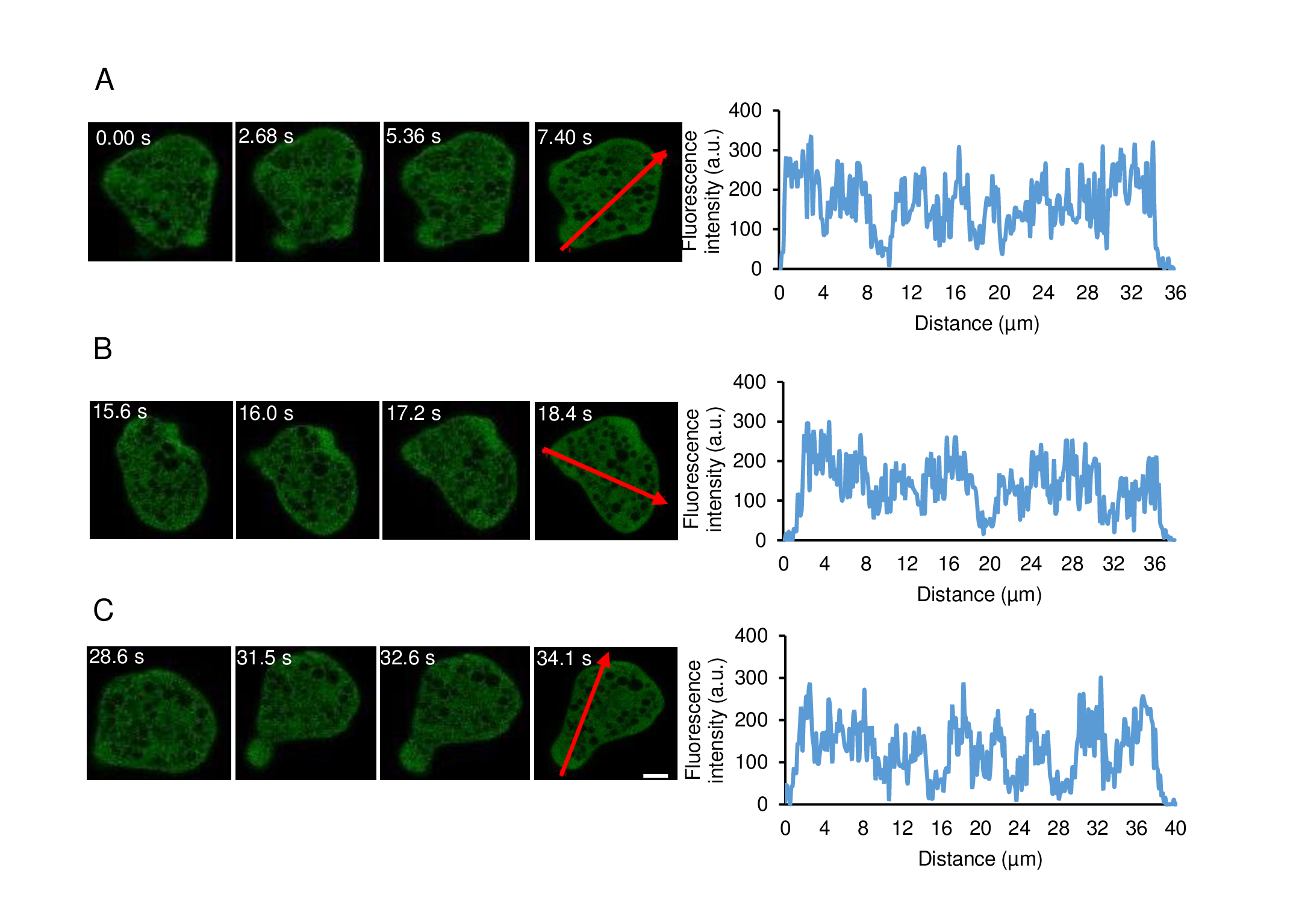

Supplement: S2 Fig — (A-C) Montage showing a time series of motile trophozoites expressing GFP in left panels. The pseudopods in different time frames have been analyzed for GFP intensity along the marked arrow line. The right panels show the fluorescence intensity of GFP across the amoebic trophozoites. (Scale bar, 10 μm). (TIF) [file ppat.1010147.s002.tif]

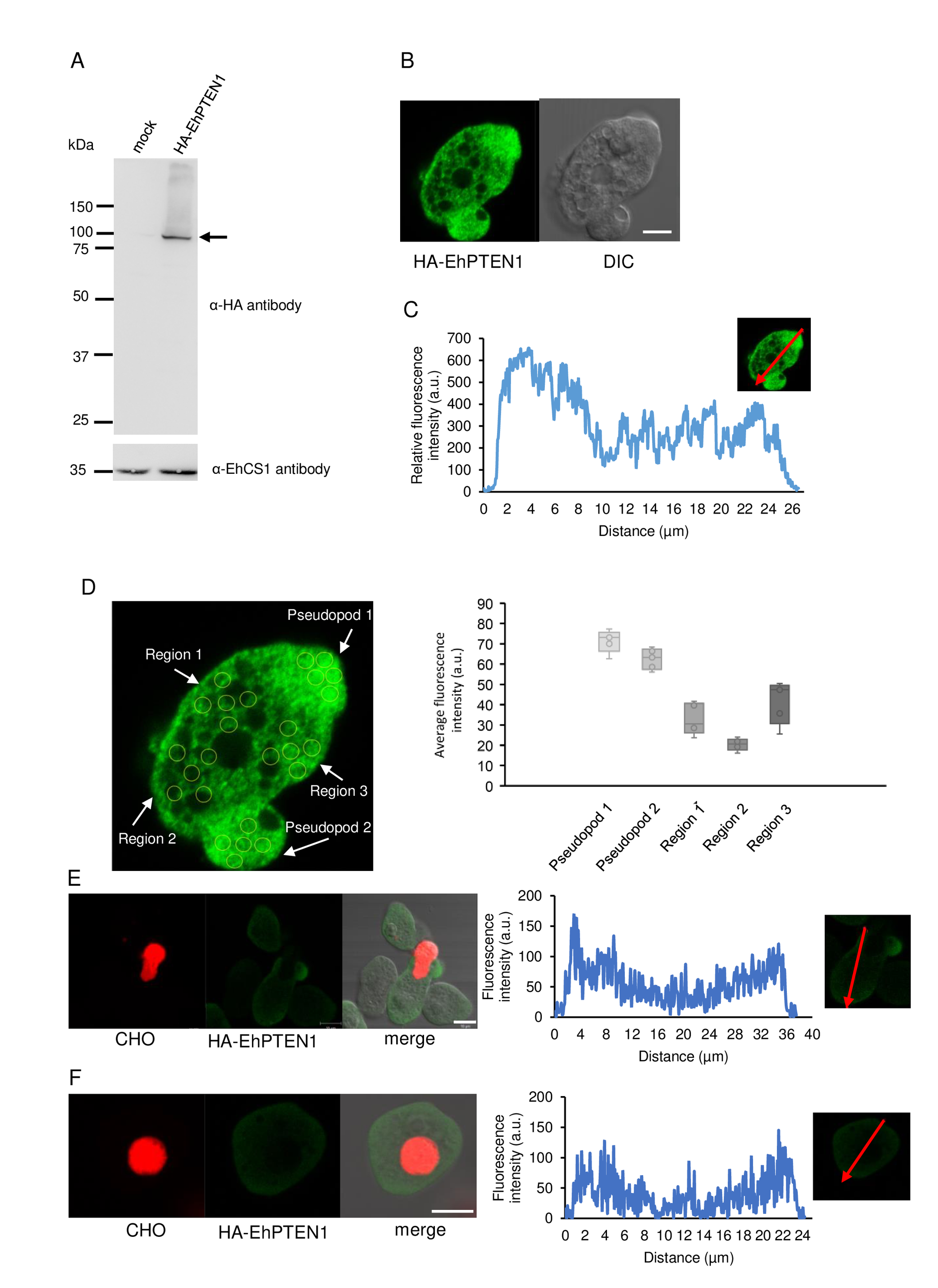

Supplement: S3 Fig — (A) Immunoblot analysis of HA-EhPTEN1 in E. histolytica transformants. Approximately 30 μg of total lysates from mock-transfected control (mock) and HA-EhPTEN1-expressing transformant (HA-EhPTEN1) were subjected to SDS-PAGE and immunoblot analysis using anti-HA antibody. EhCS1 (Cysteine synthase 1) was detected by anti-CS1 antiserum as a loading control. Arrow indicates HA-EhPTEN1. (B) Localization of HA-EhPTEN1 in a quiescent state. Immunofluorescence assay (IFA) micrographs of HA-EhPTEN1 expressing trophozoites stained with anti-HA antibody (green). (Scale bar, 5 μm). (C) The line intensity plot shows HA-EhPTEN1 intensity in pseudopods vs. cytoplasm with the distance. (D) Average fluorescent signal per pixel at different regions in the cells. Fluorescent signal per pixel in approximately 10 circular regions of interest was measured to get the average intensity per pixel. (E) Localization of HA-EhPTEN1 during initial phase of phagocytosis. HA-EhPTEN1 expressing E. histolytica trophozoites were co-cultured with CellTracker Orange-stained dead CHO cells for 15 min, fixed, and reacted with the anti-HA antibody (green). (F) Localization of HA-EhPTEN1 after phagosome maturation. HA-EhPTEN1 expressing E. histolytica trophozoites were co-cultured with CellTracker Orange-stained dead CHO cells for 30 min, fixed, and reacted with the anti-HA antibody (green). (TIF) [file ppat.1010147.s003.tif]

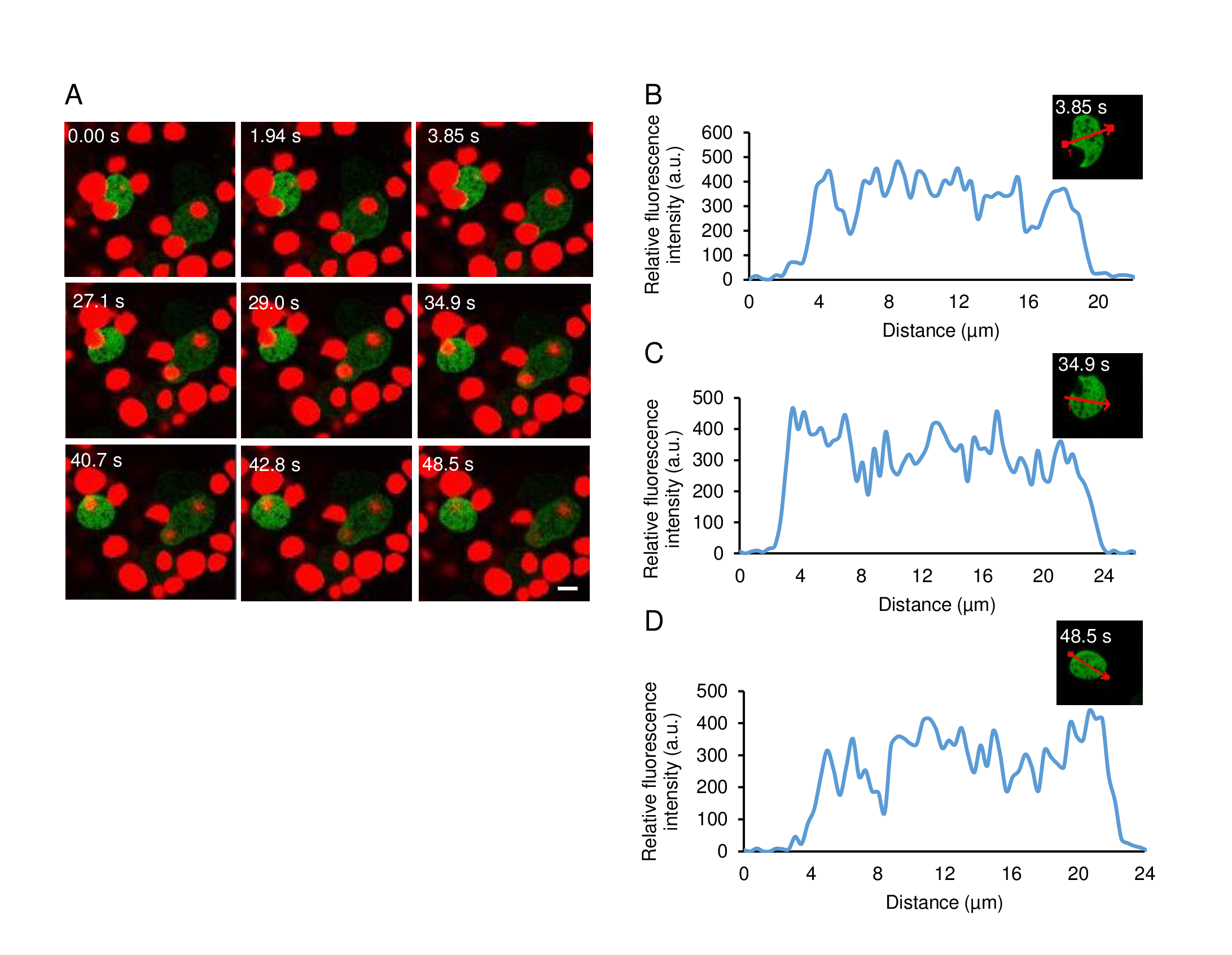

Supplement: S4 Fig — (A) Montage of live trophozoite expressing GFP ingesting pre-killed CHO cells by phagocytosis. (Scale bar, 10 μm). (B-D) Analysis of intensity of GFP across the phagocytic cup along the line drawn. (TIF) [file ppat.1010147.s004.tif]

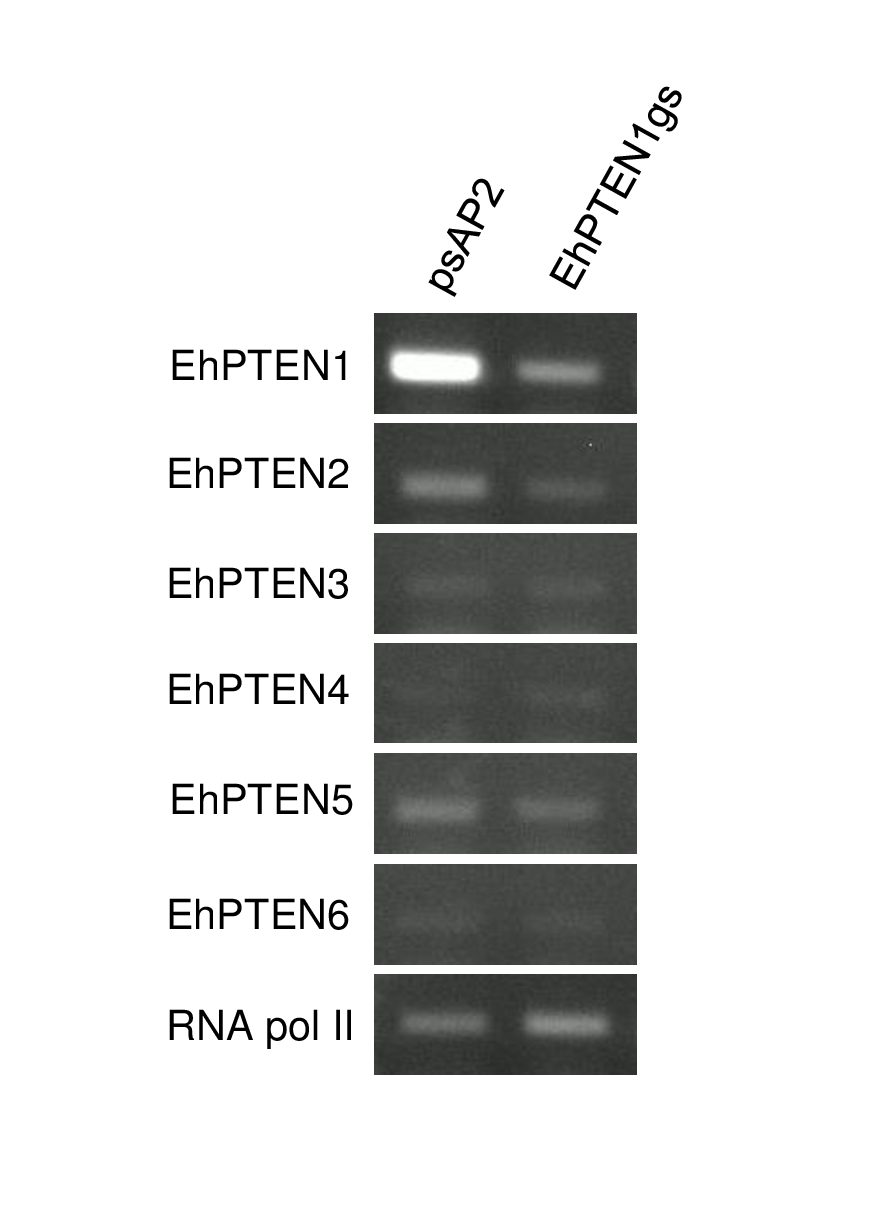

Supplement: S5 Fig — The steady-state levels of transcripts of E. histolytica PTEN isoforms and EhRNA pol II genes were measured in psAP2 mock and EhPTEN1gs transformants trophozoites. cDNA from the generated cell lines was subjected to 25 cycles of PCR using specific primers mentioned in S2 Table. RNA polymerase II served as a control. (TIF) [file ppat.1010147.s005.tif]

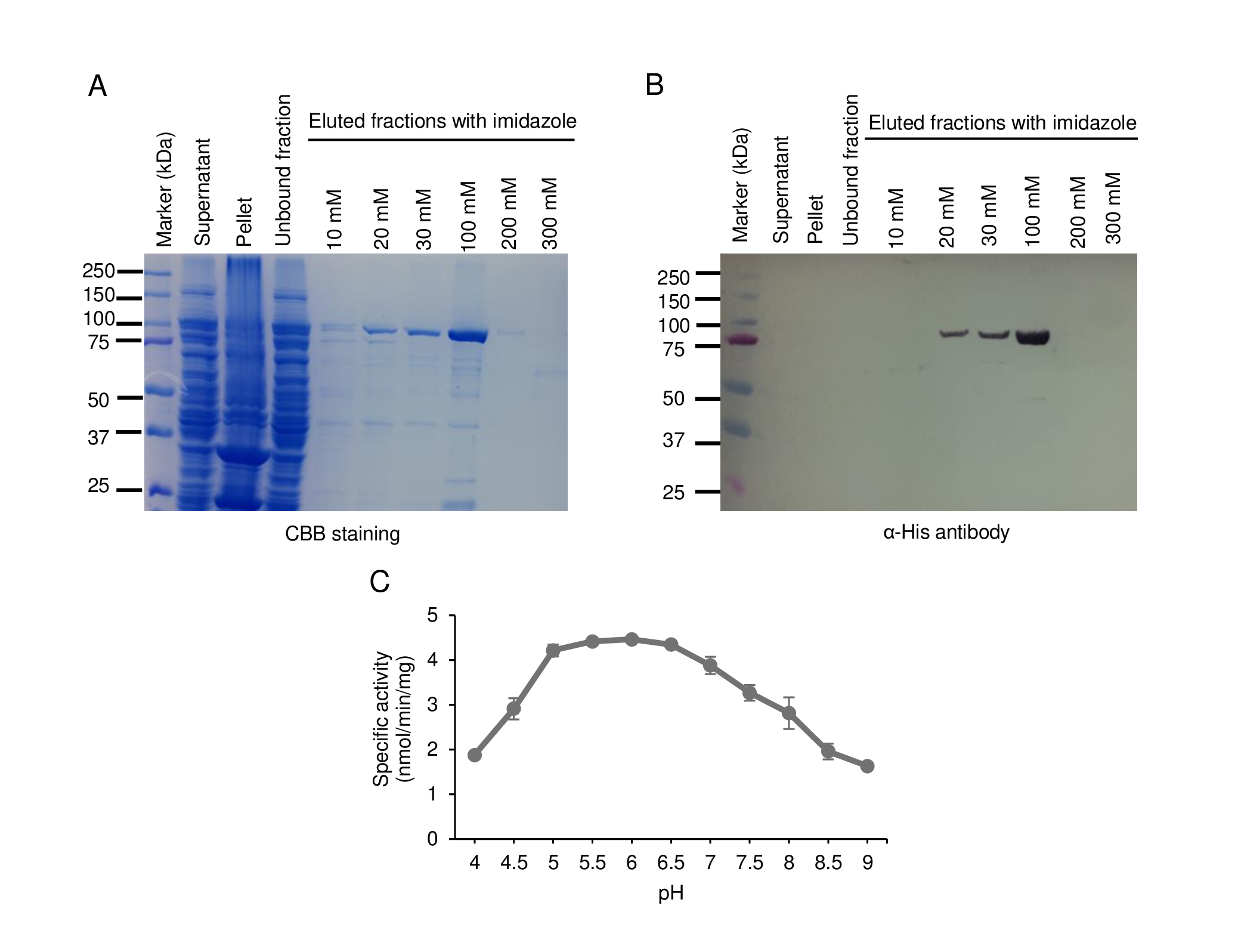

Supplement: S6 Fig — (A) Expression and purification of recombinant EhPTEN1. Protein samples at each step of purification were subjected to 10% SDS-PAGE and the gel was stained with Coomassie Brilliant Blue. (B) Immunoblot analysis of purified recombinant EhPTEN1 using anti-His-tag antibody. The recombinant EhPTEN1 in the supernatant was visualized after longer exposure. (C) Optimum pH of EhPTEN1. Enzyme specific activity of recombinant EhPTEN1 was measured at various pHs indicated in the figure. The means ± standard errors of three independent experiments are shown. (TIF) [file ppat.1010147.s006.tif]

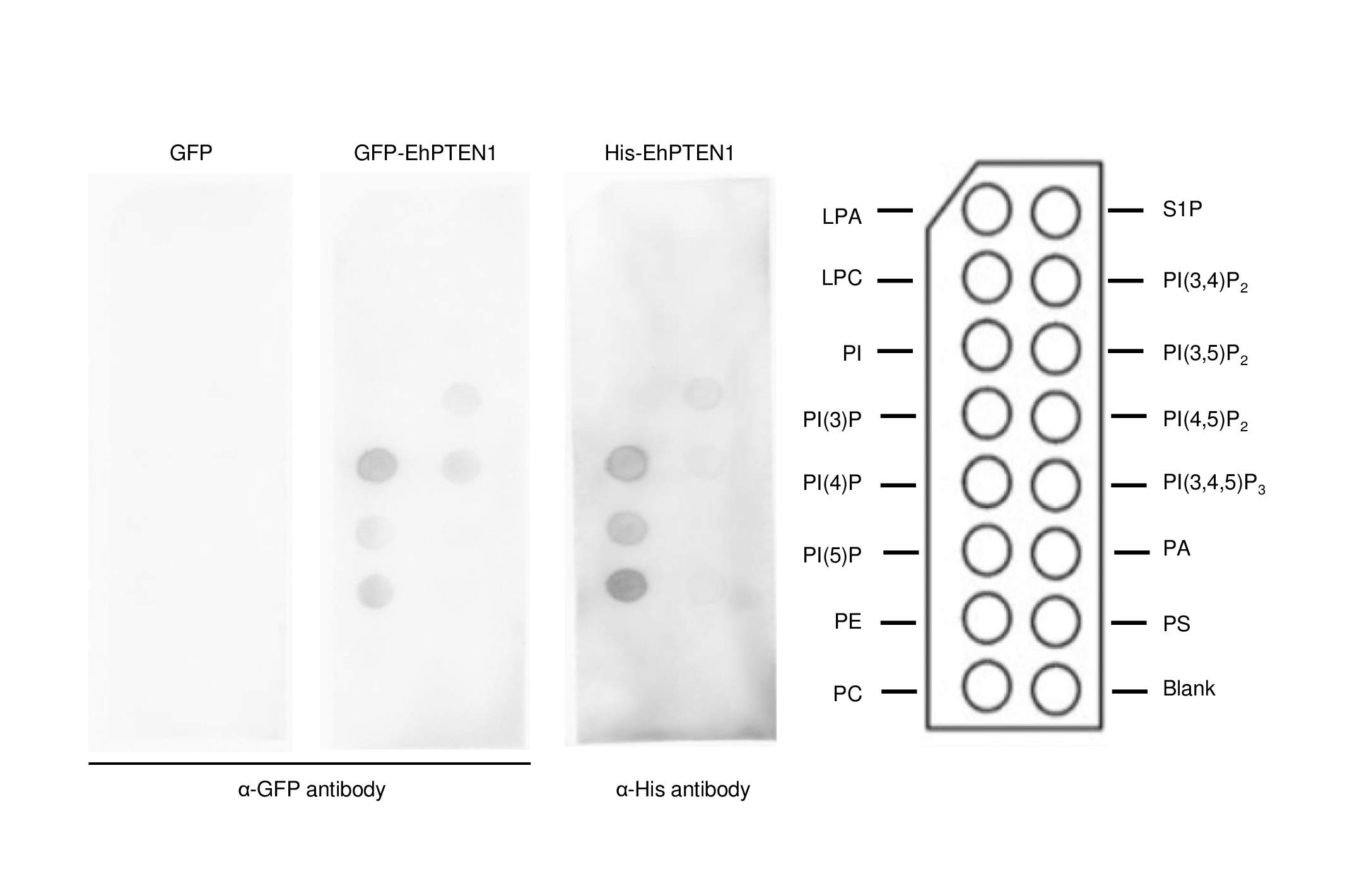

Supplement: S7 Fig — Lipid binding specificity of EhPTEN1 observed by lipid overlay assay. A panel of PIPs and phospholipid spotted in nitrocellulose membrane was incubated with total lysates from GFP-EhPTEN1 and GFP mock expressing transformants, and recombinant His-EhPTEN1. LPA, lysophosphatidic acid; LPC, lysophosphocholine; PE phosphatidylethanolamine; PC, phosphatidylcholine; S1P, sphingosine-1-phosphate, PA, phosphatidic acid; PS, phosphatidylserine. (TIF) [file ppat.1010147.s007.tif]

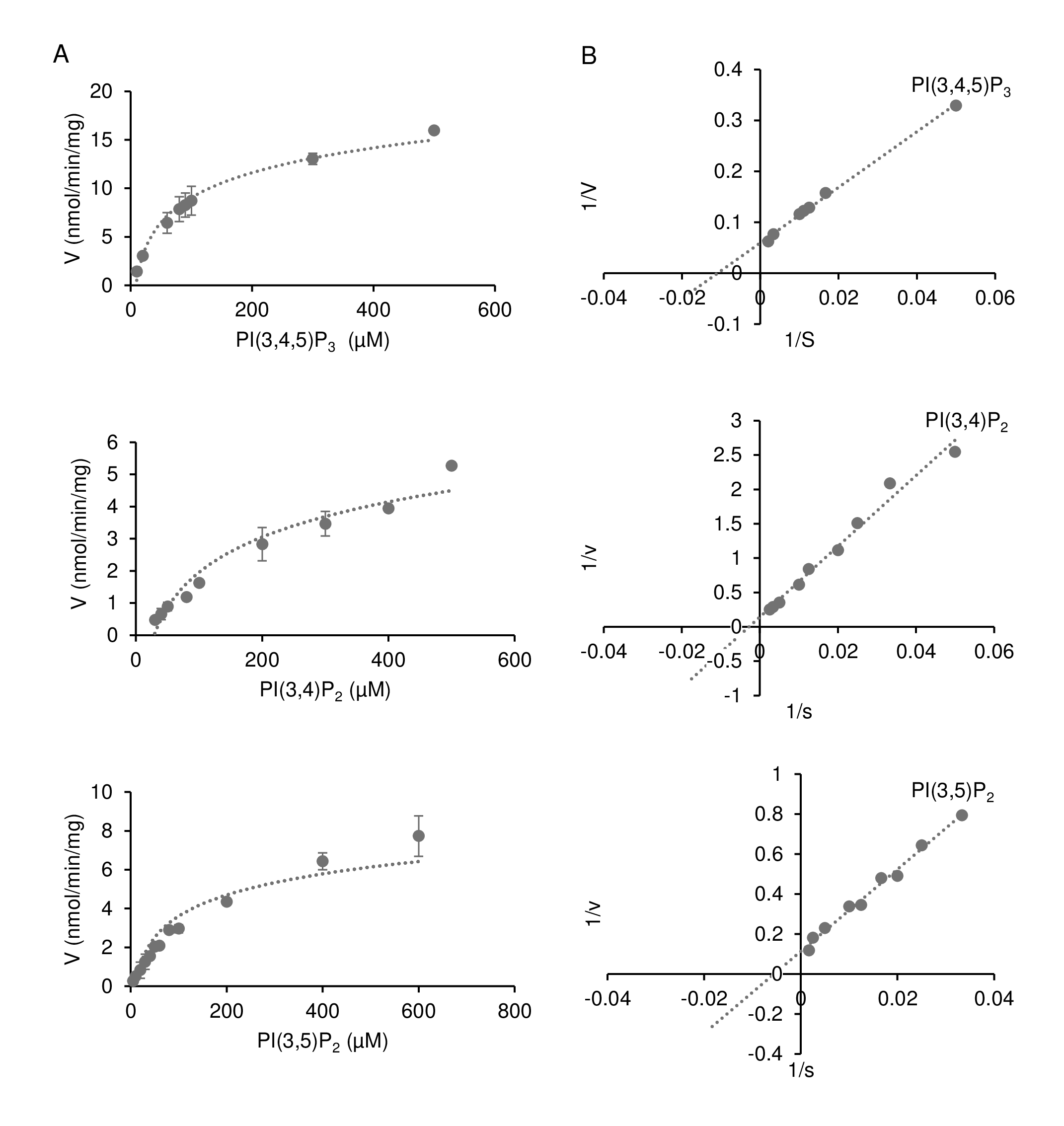

Supplement: S8 Fig — (A) Saturation Kinetics for EhPTEN1 against PI(3,4,5)P3, PI(3,5)P2, and PI(3,4)P2. Varying amount of PI(3,4,5)P3, PI(3,5)P2, and PI(3,4)P2 were mixed with EhPTEN1 recombinant protein and phosphate release was measured by a malachite green binding assay as mentioned in Materials and methods. (B) Double reciprocal plots of the recombinant EhPTEN1. The enzymatic activities were determined with various concentration of PI(3,4,5)P3, PI(3,5)P2, and PI(3,4)P2. Data are shown in means ± standard deviations of duplicate analysis. (TIF) [file ppat.1010147.s008.tif]

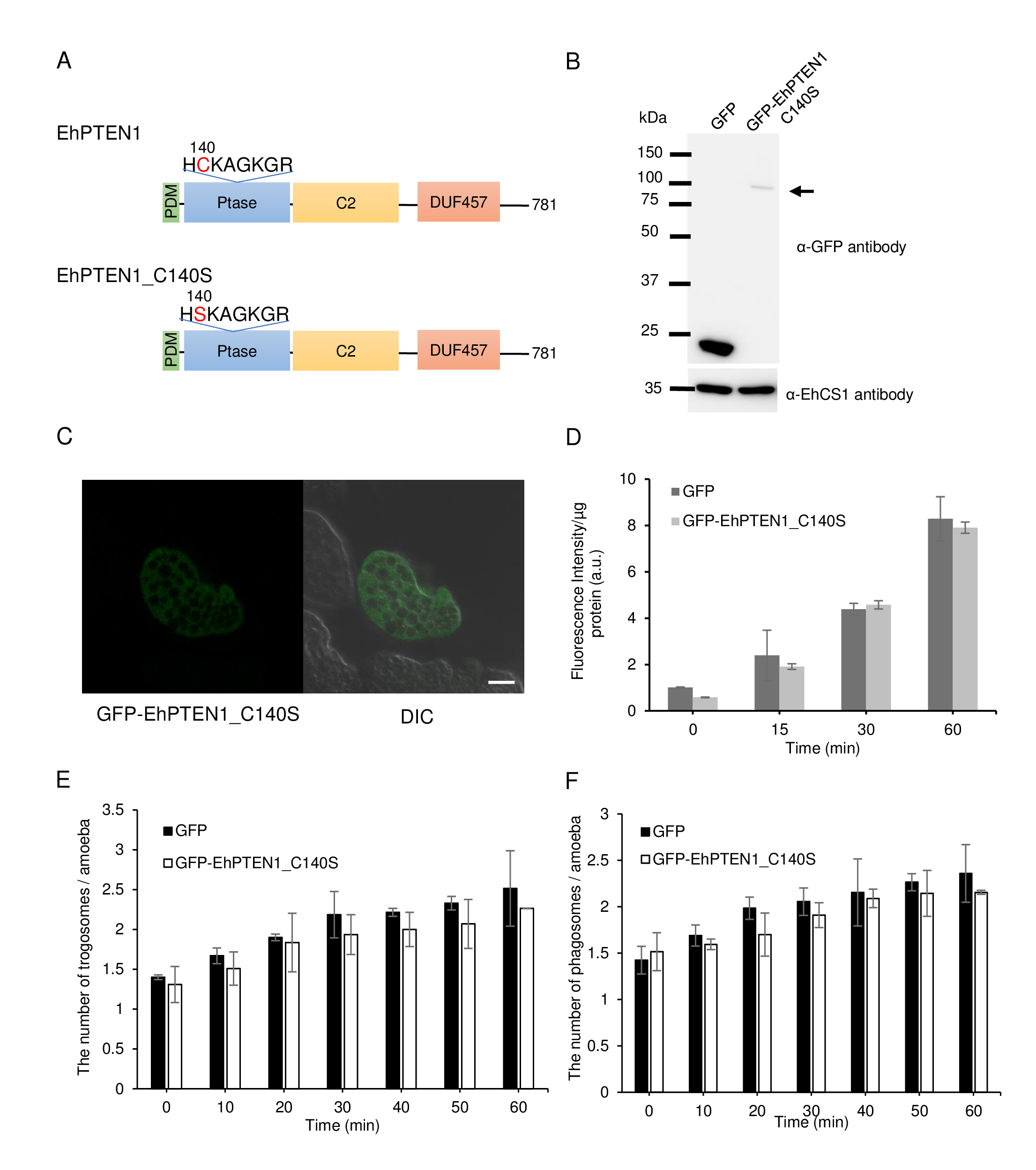

Supplement: S9 Fig — (A) Schematic representation of EhPTEN1 and EhPTEN1_C140S mutant. Amino acid residues mutated are shown in red. (B) Immunoblot of GFP-EhPTEN1_C140S and GFP (control) in E. histolytica transformants. Approximately 30 μg of total lysates from GFP mock-transfected control and GFP-EhPTEN1_C140S expressing transformant were subjected to SDS-PAGE and immunoblot analysis using anti-GFP antibody and anti-CS1 antibody. An arrow indicates GFP-EhPTEN1_C140S. (C) Localization of GFP-EhPTEN1_C140S in a quiescent state. (Scale bar, 10 μm). (D) The effect of GFP-EhPTEN1_C140S expression on pinocytosis. Trophozoites of GFP mock transfected and GFP-EhPTEN1_C140S expressing strains were mixed with RITC dextran and uptake was monitored as described in Materials and methods. Experiments were conducted twice independently and error bars indicating standard errors of two biological replicates. (E-F) Effect of GFP-EhPTEN1_C140S expression on trogocytosis (E) and phagocytosis (F). Trophozoites of GFP mock transfected and GFP-EhPTEN1_C140S expressing strains were incubated with live CHO cells (E) or heat killed CHO cells (F) that have been stained with CellTracker Orange to evaluate trogocytosis and phagocytosis, respectively. The images were captured on CQ1 as described in Materials and methods and analyzed to calculate the numbers of CHO cell-containing trogosomes or phagosomes per amoeba. Experiments were conducted twice independently in duplicates and error bars indicating standard errors of two biological replicates. (TIF) [file ppat.1010147.s009.tif]

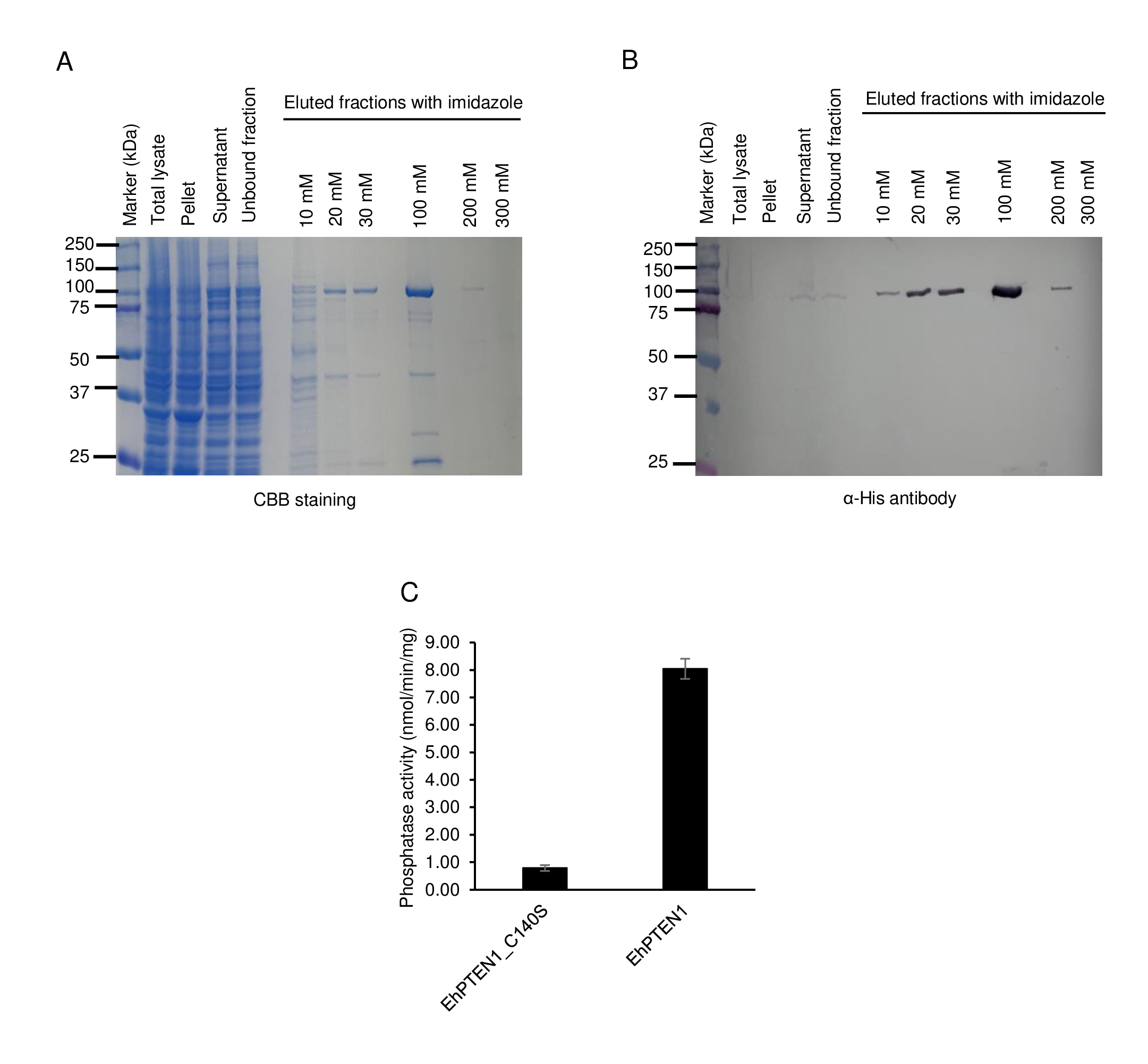

Supplement: S10 Fig — (A) Expression and purification of recombinant EhPTEN1_C140S. Protein samples at each step of purification were subjected to 10% SDS-PAGE and the gel was stained with Coomassie Brilliant Blue. (B) Immunoblot analysis of purified recombinant EhPTEN1_C140S using anti-His-tag antibody. (C) Specific activity of recombinant EhPTEN1 and EhPTEN1_C140S using 100 μM of PtdIns(3,4,5)P3 as substrate. Reactions were carried out in a volume of 25 μl for 40 min at 37°C, and terminated by the addition of 100 μl of malachite green reagent as described in Materials and methods. The absorbance at 630 nm was measured and phosphate released was quantified by comparison to a standard curve of inorganic phosphate. The mean ± S.D. of three independent experiments is shown. (TIF) [file ppat.1010147.s010.tif]

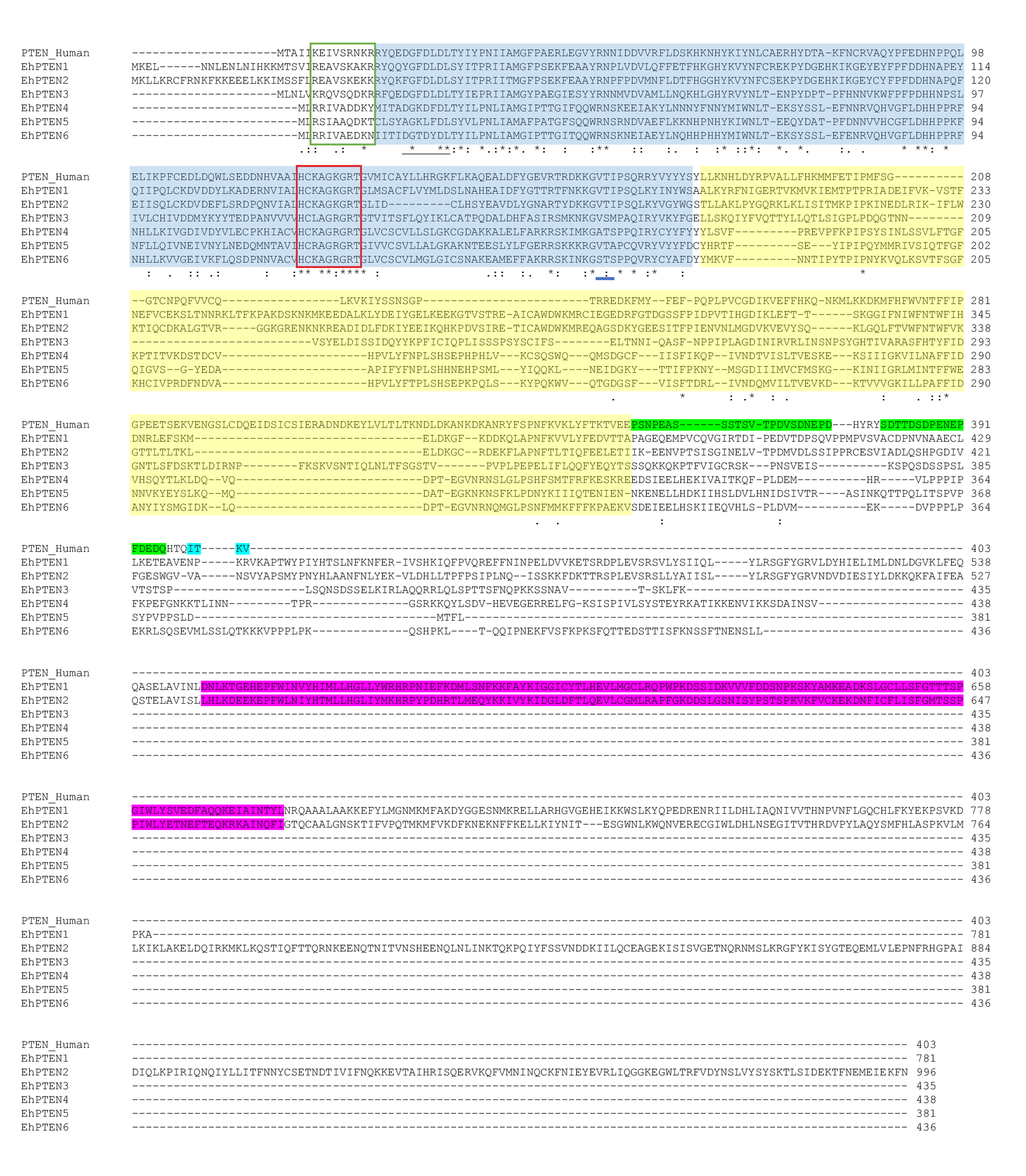

Supplement: S11 Fig — Multiple amino acid sequence alignment of human PTEN (P60484), EhPTEN1 (XP_653141.2), EhPTEN2 (XP_656021.2), EhPTEN3 (XP_655519.1), EhPTEN4 (XP_652082.1), EhPTEN5 (XP_656532.1), and EhPTEN6 (XP_654831.1) was constructed by using clustalw algorithm (http://clustalw.ddbj.nig.ac.jp). PTEN phosphatase domain and C2 domain are shown with blue and yellow backgrounds, respectively. The green rectangle corresponds to the PtdIns(4,5)P2-binding motif (PDM domain). Amino acid residues implicated for PtdIns(3,4,5)P3 catalysis are marked with a red rectangle. Cytosolic localization signal and restudies important for TI loop formation are indicated in black and blue lines, respectively. The DUF457 domain is shown in magenta background, whereas PEST and PDZ-BM are shown in green and cyan background, respectively. Note that for human PTEN, only the amino terminal part is shown. (TIF) [file ppat.1010147.s011.tif]

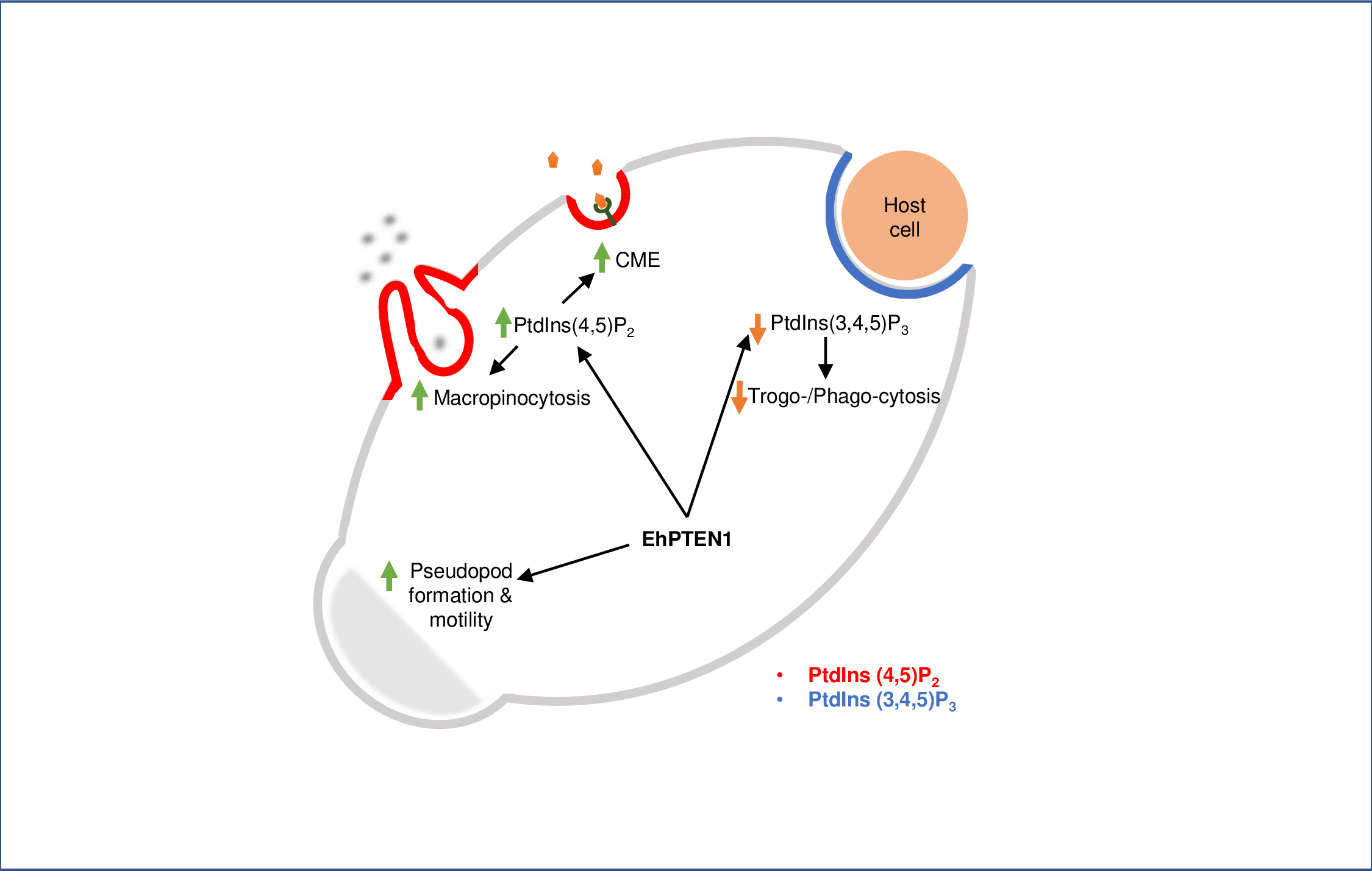

Supplement: S12 Fig — EhPTEN1 localizes mainly in the cytosol with some enrichment in pseudopod-like structures and in the early stage of trogo- and phagocytosis, probably due to changes in the lipid content of the membrane during these events. EhPTEN1 can negatively regulates trogocytosis and phagocytosis by reducing the local PtdIns(3,4,5)P3 concentration leading to the suppression of actin dependent cytoskeletal organization needed for trogo- and phagocytosis. In contrast, EhPTEN1 serves as a positive regulator of fluid-phase and receptor-mediated endocytosis in E. histolytica possibly via augmentation of PtdIns(4,5)P2 synthesis which causes recruitment of clathrin regulator and enhances formation of lipid rafts that needed for micropinocytosis. (TIF) [file ppat.1010147.s012.tif]

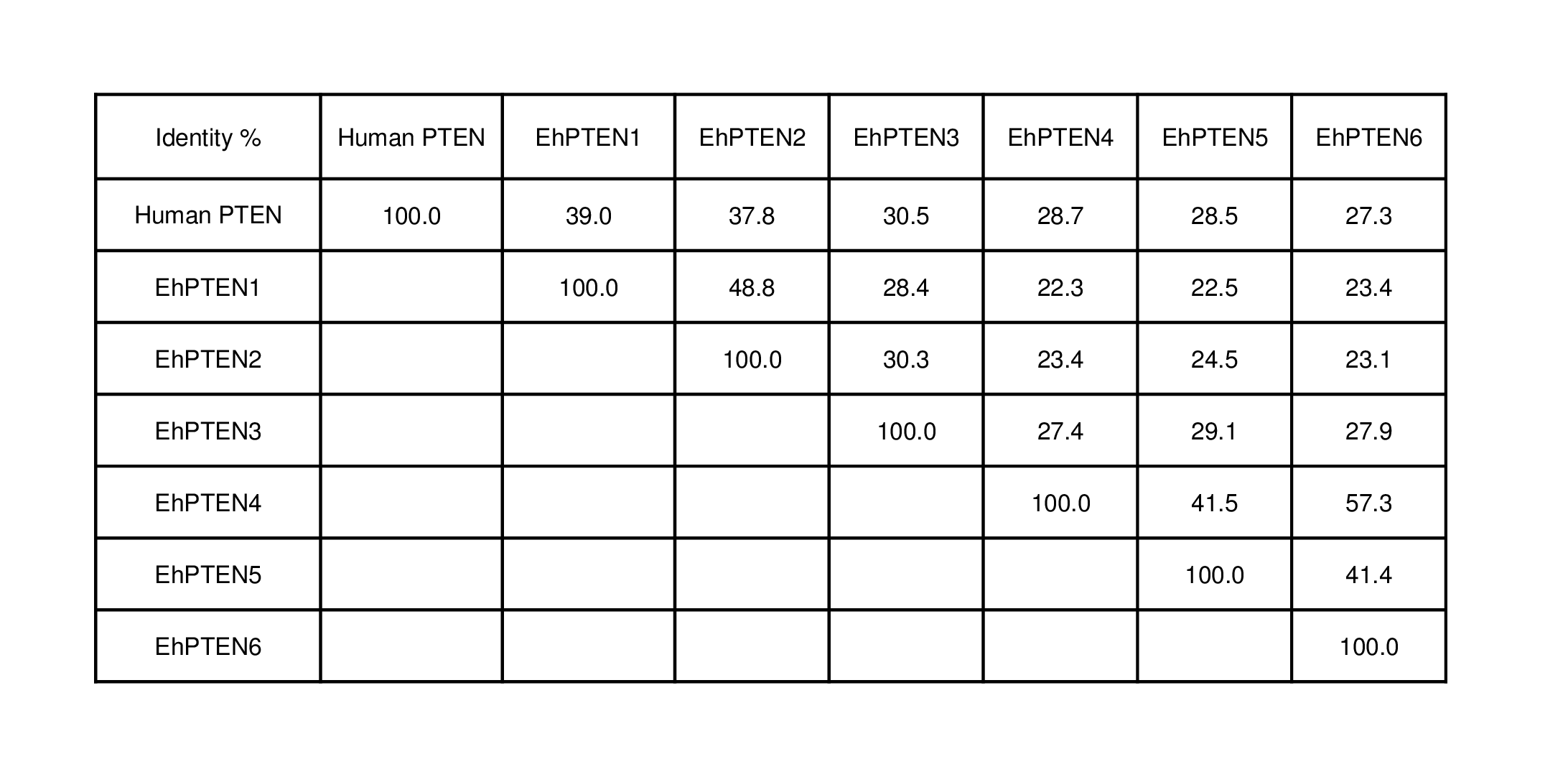

Supplement: S1 Table — (TIF) [file ppat.1010147.s013.tif]

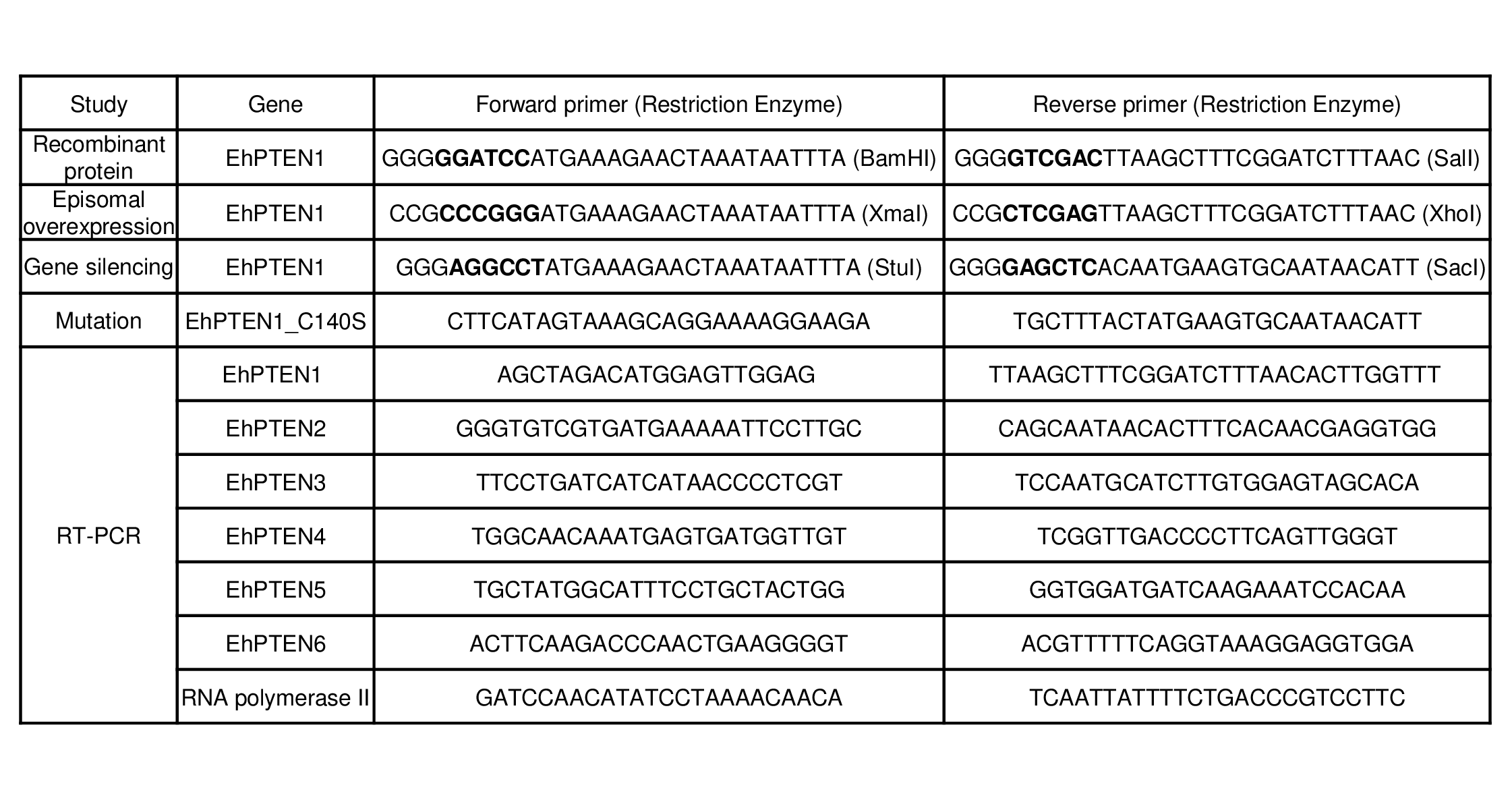

Supplement: S2 Table — Restriction site marked by bold letter. (TIF) [file ppat.1010147.s014.tif]
