# Supplementary material for: PTEN differentially regulates endocytosis, migration, and proliferation in the enteric protozoan parasite Entamoeba histolytica
Source: PLoS Pathog. 2022 May 2;18(5):e1010147. doi: 10.1371/journal.ppat.1010147 (PMC9122207; doi:10.1371/journal.ppat.1010147)
Supplement: Attachment — Submitted filename: Resopnse to reviewers.docx [file ppat.1010147.s020.docx]

We appreciate referees and editor for all constructive comments

In revision, we made the following major changes:

1. The suggested experiments using EhPTEN1 inactive mutant were performed and the results have been added as new supplementary figures (S9 Fig and S10 Fig). We have modified the text accordingly. (see below our response to comments #13 and #15).

2. Quantitation of EhPTEN1 distribution in the cell has been redone using the suggested protocol and added as S3 Fig. Panel D (see our response to comment #6)

2. Multiple sequence alignments of all PTEN isotypes from *Entamoeba histolytica* have been created (S11 Fig).

3. A working model of physiological roles of EhPTEN1 (S12 Fig) has been added to address comments #20 and #21.

4. The discussion section was shortened according to the suggestion.

Some of the suggested experiments were not performed and the reasons have been given. We sincerely appreciate your understanding of the justifications.

Please see below our point-to-point response to each comment.

**Reviewer #1 Comment #1:**

**Part II – Major Issues: Key Experiments Required for Acceptance**

The first experiment concerns the choice of the version of the isoform of PTEN which is studied according to the levels of transcription of genes corresponding to the 6 isoforms determined by bioinformatics. It has been done taking advantage of previous work from the team during description of the transcriptome of E. histolytica using microarrays technology.

- The question arises as to how, among the oligonucleotides that cover the gene sequence; the chip discriminates among the six isoforms of the transcript? (point #1)
- To reinforce the assumption of high gene expression level for the chosen PTEN, it should be of interest to inform readers about the key sequence and/or region within each transcript that discriminates the six transcripts. (point #2)
- I did a search using AmoebaDB resources (<https://amoebadb.org>) with the six gene ID and the results from multiple RNASeq experiments are: EHI_197010 is the more expressed gene, which corroborates the authors conclusion, but expression of EHI_041900 and EHI_098450 genes is also high. A better explanation of the transcription rate should be presented. (point #3)
- The further question in this part is what is the rationale to look at PTEN expression in the G3 silenced strain? There is not comment on this finding in the main text. (point #4)

**Our response:** We fully understand the concern and appreciate the suggestion.

- Point #1: The details of the DNA microarray experiments were provided in the referred papers [24-26]. Although we did not give full description of Affymetrix DNA microarray, which was the most advanced methods to understand global gene expression at the time, in short, the 11 "gene- (and isotype-) specific oligonucleotides were chosen (see below for a copy and paste of the method section of [25].). We also added an explanation in the figure legend of Fig 1B for clarification. (quote: "The expression profiles of all the genes in E. histolytica were analysed using a custom-made full genome microarray of E. histolytica on an Affymetrix platform. The array contained 9326 independent probe sets, each of which had 11 probe pairs. The probes were designed according to the genome sequence of the E. histolytica reference strain HM-1, available from the Pathema Bioinformatics Resource Center (data release 6.0).")

“The steady-state mRNA levels of PTEN isotypes in *E. histolytica* were created using the data from our previous work [24-26] where transcriptome of trophozoites of *E. histolytica* HM-1:IMSS cl6 reference strain was analyzed using DNA microarrays technology [25]. The levels of steady-state mRNA of PTEN isotypes are shown after normalization against that of RNA polymerase II.”

- Point #2: We added protein sequence alignment of six EhPTEN. (S11 Fig)
- Point #3: It should be noted that the relative abundance of each PTEN isotype may vary among the isogenic reference strain (i.e., HM-1:IMSS and G3) maintained in different laboratories. Thus, our RNA seq data may not be identical to the data deposited to AmoebaDB.

Also, to reconfirm the previous DNA microarray data, we also estimated the steady-state levels of transcripts of *E. histolytica* PTEN isoforms by RT-PCR using specific primers (listed in S2 Table) (S5 Fig). RT-PCR showed that EhPTEN1 is highly expressed in the mock transformant trophozoites, while five other PTENs are expressed at relatively low levels.

We also added the point suggested by the referee on mRNA expression levels based on RNA seq analysis. We added the following: "RNA seq data from our laboratory [76] indicate the abundance (FPKM) of six EhPTEN is as follow: EhPTEN1-6, 173.7, 25.8, 9.7, 6.7, 17.8, and 7.0 in HM-1:IMSS cl-6 transfected with HA control vector; 62.0, 11.6, 5.9, 2.0, 9.6, and 7.6 in G3 strain transfected with psAP2-Gunma mock vector (What is the unit here? Relative number of reads mapped to each gene, or just arbitrary unit? FPKM, Fragments Per Kilobase of exon per Million mapped fragments ) (page 6, line 120)

- Point #4: Small antisense RNA-based transcriptional gene silencing, used in this study, was robustly usable only in the particular clone, called G3, but not in its parental strain HM-1:IMSS. We added the point in page 11, lines 228-229 and gave proper references. We examined the PTEN expression in the G3 silenced strain to examine (and exclude) potential off-target effects on other *EhPTEN* genes. The point was added (page 11, line 231).

**Reviewer #1 Comment #2:**

In humans, PTEN is a protein of 403 amino acids that consists of five essential domains.
a-The PIP-binding domain that localizes at the N-terminal
b-The phosphatase domain containing the HCXXGXXR motif, which is the catalytic sequence in protein tyrosine phosphatases.
c-The N-terminal first 190 amino acids are homologous to the actin-binding protein tensin
d-The C2 domain that bind phospholipids in membrane independent of calcium
e-The C-terminal domain consists of 2 PEST sequences, including phosphorylated serine-threonine spots and a PDZ domain
According to Figure 1, domains a, b and d are present in EhPTEN1 and are well described in the manuscript. Unfortunately, there is no information on the tensin part inserted in the C2 domain and as well as, the consequences of the absence of the PDZ domain. In the biology of PTEN, it has been described that a truncated protein lacking the C-terminal PDZ binding motif, or proteins which extend beyond the PDZ binding motif, have been modified in the function of PTEN, including during cancer metastases (PMID: 10760291). The domains of tensin and PDZ deserve attention in this context.

**Our response:** We appreciate this valuable suggestion. We have modified the text throughout the manuscript and added explanation of the PDZ and the tensin domains. See below the modifications:

- “The homology of the first 190 amino acids in the amino terminus of PTEN to the actin binding protein tensin may suggest the involvement of PTEN in the regulation of actin dynamics.” (Page 6, line 133)
- “The human PTEN was shown to interact with several PDZ domain-containing proteins, such as membrane-associated guanylate kinase inverted (MAGI) and Na^+^/H^+^ exchanger regulatory factor (NHERF) in a PDZ-dependent manner [8,9]. Interaction of MAGI to the PTEN C-terminal PDZ domain facilitates targeting of PTEN to the plasma membrane, thereby enhancing its activity [9]. While NHERF regulates the activation of the PI3K–AKT pathway by binding and recruitment of PTEN to platelet-derived growth factor receptor (PDGFR) [8]. However, the *E. histolytica* genome apparently does not encode proteins with similarity to NHERF or MAGI, as indicated by BLAST search. Therefore, these PDZ domain binding proteins may be unique to mammals, which represents a major difference between mammalian PTEN and EhPTEN1.” (Page 7, line 141-150)

**Reviewer #1 Comment #3:**
A further look at the other potential PTEN indicate that the nomenclature proposed to distinguish them in E. histolytica proteins can lead to confusion. The absence of C2 domains indicates that two of these potential proteins lack lipid binding activity. Are these bona fide PTEN? In humans, variations of PTEN derive from the same loci (PMID: 24768297, PMID: 23744781) after the start of alternative translation. In E. histolytica, isoforms 1 and 2 appear to be homologous (48.8% identity in Table S1). I have run the CLUSTALW program with the mRNA sequences of both genes and there is 64% identity between them; moreover, in isoform 2 the DNA sequence presents introns. This is important because in this work it is shown that E. histolytica retained at least 4 isoforms of PTEN, many more than in the other species studied. Perhaps a more restrictive nomenclature should help avoid future confusion (Point #1). Additionally, more in-depth analysis of other PTEN isoforms could be provided by at least benefiting from transcriptome analysis, along with proteomic analyzes from the team and other groups, to convince readers that these proteins exist (Point #2).

**Our response:** We appreciate the comment.

Point #1: It remains to be the issue of debate how we should designate and genes that belong to a gene family before enzymatic and biochemical evidence of individual proteins is demonstrated. Demonstration of enzymatic activity and lipid binding activity of all six PTEN isotypes is beyond the scope of this study. However, six proteins (EhPTEN1-6) described in this study are considered to be authentic PTEN homologs because key important domains and residues, such as the Ptase (Phosphatase tensin-type domain) and key catalytic residues (H-C-K/R-A-G-K-G-R) needed for PTEN activity, are well conserved in all six isotypes, although EhPTEN5 and EhPTEN6 lack C2 domain. That is our justification to tentatively designate the proteins EhPTEN1-6. However, if the referee still thinks it is not restrictive enough nomenclature, it is also acceptable to designate them as EhPTEN1, EhPTEN-like proteins 2-6.

Point #2: As far as we are aware, there is no proteome data available to support the presence of other proteins. Our proteome data is basically restricted to phagosomes. However, our preliminary immunoprecipitation of EhPTEN1 indicates interaction with EhPTEN2, detected by MS analysis (data not shown; not included in the manuscript), reinforcing at least EhPTEN2 is expressed in our HM-1:IMSS.

**Reviewer #1 Comment #4:**
Regarding cell biology experiments like live cell imaging and image analysis, several shortcomings deserve the author's attention: It is mandatory to describe the experimental setting for live cell imaging, microscopy approaches and software implementations. For example, which confocal plane, which path for image analysis, what is CQ1? What references and what algorithms are used in the CellPathfinder software. As like other technical approaches, the reader needs references for compounds, companies, and step-by-step methods. May I respectfully tell the authors to look at the article PMID: 32111929 in the topic PTEN, which, in my opinion, describes the imaging and image analysis approaches very well.

**Our response:** We appreciate the suggestions for the reference (PMID: 32111929). For clarity, we added the description of the materials and methods for live cell imaging. Also, we included one reference in which CQ1 was exploited with some modifications explained as follow:

- “Live images were captured on Zeiss LSM780 confocal microscope (Carl-Zeiss, Oberkochen, Germany) using a 63× oil immersion objective with default settings on the time series mode and analyzed by ZEN software (Carl-Zeiss). For live imaging of trogocytosis and phagocytosis, approximately twenty cells were analyzed per each cell line in an experiment of three independent experiments. GFP-EhPTEN1 recruitment was estimated by quantitating the average fluorescent intensity per pixel across the line drawn over the cell or that of 11-13 regions of interest on the pseudopods and other parts of the cell.” (Page 23, line 513-519)
- “We quantified trogocytosis and phagocytosis efficiency using an image cytometer as described previously [76]. Briefly, trophozoites of *E. histolytica* were incubated in BIS containing 10 µM CellTracker Blue (Thermo Fisher) at 35.5°C for 1 hr.” (correction shown underlined) (Page 24, line 533)
- “The images of trogocytosis and phagocytosis were captured on a Confocal Quantitative image cytometer CQ1 (Yokogawa Electric Corporation, Tokyo, Japan) using a 20× objective every 10 min for 1 hr. Five fields each per well for three wells were chosen for image analysis. Images on five planes with 2 μm intervals in Z axis were obtained to calculate the volume of the ingested CHO cells using three‐dimensional reconstructed images. For analysis of motility, images of approximately 400 cells were captured from each well and analyzed using CellPathfinder software (Yokogawa Electric Corporation, Tokyo, Japan) according to the manufacture’s protocol. The protocol for quantitative analysis of the images was optimized based on the cell morphology and fluorescence intensity to identify *E. histolytica* trophozoites and CHO cells in each field.” (Page 24, line 541-550)

**Reviewer #1 Comment #5:**
I understand the difficulties of studying gene expression and protein abundance in E. histolytica due to the lack of genetic tools to obtain stable phenotypes. Here, the overexpression of PTEN is obtained by the insertion of two hybrid genes: GFP-PTEN (useful for monitoring GFP in live cell imaging) and HA-PTEN (useful for monitoring PTEN in immunofluorescence of fixed using HA antibodies). These two constructs were introduced into the strain E. histolytica HM-1: IMSS c16.

Enrichment of GFP-EhPTEN1 in the pseudopod of living trophozoites has been observed. Their shifts in speed of displacement were 0.54 ± 0.09 µm / sec (mean ± S.D.), which was significantly greater than that of transformants expressing control GFP (0.27 ± 0.08 µm / sec) ~roughly 2 times.

The effect of repressing the expression of the EhPTEN1 gene was obtained by inserting a silencing construct into the G3 strain, which was shown, at the start of work (FIG. 1B), expressing the gene encoding PTEN twice. In strain G3, transformed by the silencing construct targeting EhPTEN1, the displacement speed is reduced (0.16±0.07 μm /min) compared to the mock control (0.44±0.08 μm /min) ~roughly 2,7 times. If we compare the two mock controls: GFP (0.27 ± 0.08 µm / sec) and G3 (0.44±0.08 μm /min) the change is 1.6.

- I could be wrong, but how do you consider these discrete changes as important in the regulation of cell motility? (point #1)

In addition, the strains have different basal level of expression of the PTEN gene. For these reasons, the conclusion regarding cell motility seems weak. Inhibition of PTEN in Dictyostelium alters cell morphology (PMID: 29602807); as well as the nature of their displacements.

- Further analysis of video microscopies of PTEN-deficient cells, to examine the morphology and direction of trajectories, may help to strengthen this data. Moreover, in some cases, the inhibition of Pi3K can "rescue", at least in part, the phenotype deficient in PTEN. (point #2)

**Our response:** We appreciate the valuable comments and suggestions.

- Point #1: The average speed of the G3 mock control strain in our study is within the range that were previously demonstrated (PMID: 32975360 and PMID: 32707151). However, the difference in motility between the two mock control stains (HM-1: IMSS c16 and G3 stains) could be due to intrinsic differences including cell size, plasma membrane rigidity, and drug concentrations cells are exposed to. Related to the last point, cell lines that express either GFP mock and GFP-EhPTEN1 were maintained in the presence of 20 μg/ml G418, while *EhPTEN1* gene silenced and psAP2-Gunma mock vector transfected G3 strain were maintained with 10 μg/ml G418.

As explained above, the effects of expression repression by gene silencing can be observed ONLY in G3-derived transformants; in this case we had to compare phenotypes in G3-derived gene silenced strain in which target gene is silenced against G3 transformed with mock vector (also see our response to Reviewer 1 Comment #1 Point #4). In contrast, the effects of overexpression of a protein of interest (GFP-EhPTEN1) was observed in HM-1:IMSS (wildtype)-derived GFP-EhPTEN1 overexpressing strain and the parental strain (HM-1:IMSS). There are a number of differences such as in gene expression profiles and behaviors between HM-1:IMSS and G3. However, we believe we have clearly demonstrated direct causal association (i.e., by either silencing of *EhPTEN1* or overexpression of GFP-EhPTEN1) between EhPTEN1 and cell motility.

- Point #2: Regarding the morphological changes, we also noticed that the area of the matrix surface adherent part of *EhPTEN1*-gene silenced cells is larger than psAP2 mock strain (data not shown). Statistical significance, however, of the changes was not proven, and the data were not included in the manuscript. However, this observation may partially explain how surface adhesion and motility correte, and reinforces that the increment in the cell adhesive area caused by EhPTEN1 gene silencing and the increase in motility are probably associated. We are also planning to examine direction of trajectories once 3D colon organoid model is successfully constructed (in progress in collaboration with Institut Pasteur and University of Washington). In future, we will report the trajectory analysis using the 3D model and updated tracking software under development at Institut Pasteur. The suggested rescue experiment by inhibition of PI3K is an attractive addition in our study, but we are afraid the experiment does not directly strengthen our claim on the physiological roles of EhPTEN1, and have decided not to perform the experiment. We sincerely hope the reviewer kindly accept the justification.

**Reviewer #1 Comment #6:**
Immunofluorescence on fixed trophozoites overexpressing HA-EhPTEN1 (using anti-HA antibodies) led the authors to say that HA-EhPTEN1 was localized in the cytoplasm at steady state and enriched in pseudopodia (Fig S3B and S3C). Here, the calculations were carried out along a line crossing the cell, but the choice of this line is biased because it appears that similar fluorescence levels are also observed in the back part of the cells. The statement is misleading. A better approach should be to determine fluorescence (pixels) in several regions of interest inside the cells and then compared these values (Point #1). The speed of movement of these cells has not been determined (Point #2).

**Our response:** We fully understand the concern and appreciate the suggestion.

- Point #1: We have, accordingly, redone the analysis by measuring fluorescent signal per pixel at different regions in the cells. We have added the new analysis and added the data in S3 Fig. panel D, and modified the text as follow.“Similarly, immunofluorescence imaging of HA-EhPTEN1 overexpressing trophozoites using anti-HA antibody revealed that HA-EhPTEN1 was localized in the cytoplasm in steady-state, and enriched in pseudopods when the fluorescence signal across the line traversing the cell was measured (S3C Fig). HA-EhPTEN1 localization was also evaluated by measuring the fluorescence signal in the different portion of the cell, confirming that HA-EhPTEN1 is highly concentrated in the pseudopods compared to other parts of the cell (S3D Fig).” (Page 8, line 172-177)
- Point #2: We apologize for the mistake. We have removed the statement related to the speed of HA-EhPTEN1 as the data has not been provided in this study. (Page 18, line 406)

**Reviewer #1 Comment #7:**
7. My last comment refers to the fact that PTEN is a PI 3-kinase antagonist; the two enzymes having important contrasting roles in metabolism and cell growth. Additionally, some articles examine the role of Pi3K in E. histolytica during phagocytosis and human cell death (eg, PMID: 25246714, PMID: 9317033). I am surprised that the Pi3K antagonistic role of PTEN was not considered in this work. At least this question could be raised in the discussion chapter.

**Our response:** We appreciate this valuable suggestion. We have addressed this point in the discussion as follow:

- “Furthermore, it was previously reported that treatment with wortmannin, the PI3K inhibitor, impaired phagocytosis of several particles including bacteria, mucin-coated beads, and hRBCs by *E. histolytica* trophozoites [78,41]. These data may be consistent with the mutual antagonistic role of PTEN and PI3K.” (Page 16, line 347)

**Comment #8 (Reviewer #2 Comment #1):**

Many experimental details that are critical for rigor and reproducibility are missing (mainly related to the number of cells/images analyzed and the number of biological repeats performed). These details are necessary in order to be able to assess the robustness of the conclusions of these studies. There are four main points to address: (1) It is critical to add information on how many cells were analyzed for the imaging experiments and how representative the examples are of the population level phenotypes. Many of the conclusions appear to be based on the analysis of a single example cell shown in the figures (for example, the localization of the tagged proteins during trogocytosis and phagocytosis in Fig 3 and Fig 4).

**Our response:** We appreciate this valuable suggestion. We have added more information regarding live imaging experiment in the materials and methods section as follow:

“For live imaging of trogocytosis and phagocytosis, approximately twenty cells were analyzed per each cell line in an experiment of three independent experiments. GFP-EhPTEN1 recruitment was estimated by quantitating the average fluorescent intensity per pixel across the line drawn over the cell or that of 11-13 regions of interest on the pseudopods and other parts of the cell.” (Page 23, line 515)

**Reviewer #2**

**Comment #9 (Reviewer #2 comment (2)):**

Heterogeneity in tagged protein expression levels is also evident in the movies and still images (meaning there is one bright cell that was analyzed, and many dimly expressing cells in the surrounding imaging field), but this heterogeneity is not addressed or accounted for.

**Our response:** We agree to the point that heterogeneity was observed in GFP-EhPTEN1 and GFP expression in the respective transformant population. However, even in the cell that expressed GFP-EhPTEN1 only dimly showed the accumulation of GFP-EhPTEN1 in the pseudopod like structure and at the initial stage of trogo-/phago-cytosis (S4 Movie). We added this point as follow:

“Although the levels of expression of GFP-EhPTEN1 and GFP varied in the respective population, the accumulation of GFP-EhPTEN1 in the pseudopod like structure at the initial stage of trogocytosis and phagocytosis (S4 Movie) was observed even in the cell that expressed GFP-EhPTEN1 at low levels (showing only dim fluorescence).” (Page 8, line 168-172)

**Comment #10 (Reviewer #2 comment (3)):**

In many of the experiments for which statistical analysis was performed (Fig 2d, 5, 6, 7d, 8, 9, 10, 11), the explanation of what is plotted is vague. In these cases, the number of biological repeats that were performed is stated, but it is not clearly explained whether the figures reflect one repeat (i.e., the error bars reflect technical replicates), or the average of all of the biological repeats (i.e., the error bars reflect biological replicates.

**Our response:** We apologize if the description was unclear and led to any confusion. All figures showed the average of three biological replicates unless otherwise stated. For clarification, we rewrote the figure legend as follow (correction shown underlined):

- Fig 2 : “(D) Cell motility of GFP and GFP-EhPTEN1 transfected strains. Time-lapse images of the transformant trophozoites were collected every sec for 5 min using CQ1 and 30 cells were selected randomly for analysis by CellPathfinder software. The average of three independent experiments is shown. Statistical significance was examined with Dunnet test (**P< 0.05).” (Page 31, line 703)
- Fig 5: “Error bars indicate standard deviations of two biological replicates.” (Page 32, line 730)
- Fig 6: “Error bars indicate standard deviations of two biological replicates.” (Page 33, line 739)
- Fig 7: “(D) Cell motility of psAP2 mock transfected and EhPTEN1 gene silenced strains. The indicated transformant trophozoites were pre-stained with CellTracker green and time-lapse images were collected every sec for 2 min using CQ1 and 30 cells were selected randomly for analysis by CellPathfinder software. The experiments were performed three times independently. The average of three independent experiments is shown. Statistical significance was examined with Dunnet test (**P<0.05).” (Page 33, line 751)
- Fig 8: “Error bars indicatite standard deviations of three biological replicates.” (Page 34, line 770)
- Fig 9: “Error bars indicate standard deviations of three biological replicates.” (Page 34, line 778)
- Fig 10: “Error bars indicate standard deviations of three biological replicates.” (Page 35, line 789)
- Fig 11: As already stated above: “The means ± standard deviations of three independent experiments performed in duplicates are shown.” (Page 35, line 796)

**Comment #11 (Reviewer #2 comment (4)):**

It is not clear how many cells were analyzed for the CQI analysis or how cells were selected for quantitative analysis. (Point #1)

The localization data for the EhPTEN1 are not strong enough to support the authors’ conclusions about where these proteins localize during trogocytosis and phagocytosis. The imaging quality/resolution is very low, and it also appears that in some cases, what appears to be dynamic localization of the proteins over time is actually just a random change in the plane that was imaged. Higher resolution images from immunofluorescence analysis capturing many cells at various stages of trogocytosis and phagocytosis would be much more conclusive, particularly if combined with co-localization analysis of actin or other relevant proteins. These types of studies would be the strongest if the data clearly reflect assessment of many individual cells. (Point #2)

**Our response:** We fully understand the concern and appreciate this valuable suggestion.

- Point #1: We have revised Method section, and provided more detailed description of the methods used for analysis using CQ1. We have added the following statement in the method section:

“The protocol for quantitative analysis of the images was optimized based on the cell morphology and fluorescence intensity to identify *E. histolytica* trophozoites and CHO cells in each field” . Regarding the cell number for CQ1 analysis, as addressed above, we added the number of cells that have been analyzed (see our response to Reviewer #1 Comment #4).

- Point #2: Immunofluorescence analysis, in which fixed cells and antibodies and a confocal laser scanning microscope (LSM) are used, usually provides higher resolution as compared to time lapse imaging. However, live imaging is, with a tradeoff for resolution, suitable to determine the specific timing in a course of trogocytosis and phagocytosis. We also performed IFA analysis using HA-EhPTEN-expressing trophozoites during phagocytosis (S3E and S3F Fig), which showed very similar phenotypes (recruitment of EhPTEN1 to the pseudopods) to GFP-EhPTEN1-expressing transformant. Also, as addressed in our response to the previous comment (our response to Reviewer #2 Comment #9), almost all cells in the population, largely irrespective of expression levels, showed similar phenotypes by live imaging analysis. We sincerely hope that the reviewer would kindly understand how hard it is to capture the process of trogo- and phagocytosis in "many" cells. This is why we have presented only representative images/movies. Investigation on the kinetics of colocalization of (or lack of colocalization of) actin, actin related proteins, calcium binding proteins, protein kinases, and phosphoinositides, with EhPTEN1 on the phagocytic and trocytic tunnel, cup, and phagosome/trogosome during phagocytosis and trogocytosis is an exciting new area, but we are afraid the topic is far above the scope of the present study. We are planning to address this important subject in future.

**Reviewer #3**

**Comment #12 (Reviewer #3 Comment 1):**

PTEN is one of the enzymes that is involved in PI3P levels and is part of the system that maintains an intricate balance of all PIPs. It is, therefore, important to include a summary of our understanding of different PIPs and the proteins that participate in E. histolytica (Point #1). The authors should indicate the effect of increasing or decreasing the amount of PTEN to levels of other PIPs and experimentally determine the levels of these metabolites after overexpressing and down-regulating the enzyme. (Point #2)

**Our response:** We appreciate the valuable suggestions.

- Point #1: For the current understanding of PIPs and PIP-metabolizing enzymes from *E. histolytica,* the following review [20] should be referred, which was sited.
- Point #2: Determining PIPs levels in the cell lines EhPTEN1 is either overexpressed and gene silenced may not demonstrate changes in the intracellular global (not local) PIPs levels. This is because changes in the local PIPs concentration in the EhPTEN1 overexpressing or gene silenced transformants are likely subtle. In addition, from our previous experiments, anti-PtdIns(3,4,5)P_3_ antibody did not work well in *E. histolytica*. In addition, we are currently unable to carry out lipidomic analysis required to accurately determine intracellular PIP concentrations. Thus, we regret to say we have decided not to address this point. Alternatively, we demonstrated lipid phosphatase activity of the recombinant EhPTEN1 enzyme and its PIP specificities to provide evidence of the identified EhPTEN1 being PtdIns(3,4,5)P_3_ 3-phosphatase.

**Comment #13 (Reviewer #2 Comment 2):**
In order to demonstrate that the enzymic activity of PTEN is responsible for its’ phenotype, it is important to overexpress inactive enzyme (mutated) and then observe the phenotype of the cells.

**Our response:** We fully understand the concern and appreciate the suggestion. We have created a line that expresses GFP-EhPTEN1_C140S and added new results of phenotypic analysis as a new supplementary figure (S9 Fig). Expression of the inactive mutant caused no significant change compared to GFP mock control, which supports our argument regarding this issue. However, the expression level of GFP-EhPTEN1_C140S is lower than that observed in control and catalytic active GFP-EhPTEN1 which may lead to only insignificant changes. However, we cannot totally rule out the possibility that lack of phenotypes by overexpression of GFP-EhPTEN1_C140S is due to low level of expression. We have modified the text to address this point in S9 Fig as follow:

- In Methods

**“Site directed mutagenesis of EhPTEN1**

Overlapping primers were designed to replace cysteine at amino acid 140 of the pEhExGFP-EhPTEN1 and pCOLD1-EhPTEN1 plasmids with serine. The primer sets used are listed in S2 Table. PCR reaction was carried out using 0.2 μM/μl each of the primers, 25 μl of PrimeSTAR Max DNA polymerase (Takara Bio Inc, Shiga, Japan) and pEhExGFP-EhPTEN1 or pCOLD1-EhPTEN1as a template. The PCR parameters were: incubation at 95°C for 1 min; followed by 30 cycles of denaturation at 98°C for 10 sec, annealing at 55°C for 15 sec, and elongation at 72°C for 50 sec; and a final extension at 72°C for 30 sec. The PCR fragment was evaluated by agarose gel electrophoresis and introduced into DH5α competent *E. coli* cells by heat shock at 42°C for 45 sec. Bacterial colonies containing the plasmid that encodes mutated EhPTEN1 (pEhExGFP-EhPTEN1_C140S and pCOLD-1-EhPTEN1_C140S) were selected from LB plate containing 100 μg/mL ampicillin by DNA sequencing of the plasmids. The pEhExGFP-EhPTEN1_C140S plasmid was introduced into the trophozoites of *E. histolytica* HM-1:IMSS cl6 strain as described previously [81]. Transformants were initially selected in the presence of 1 μg/ml G418 until the drug concentration was gradually increased to 20 μg/ml. The pCOLD-1-EhPTEN1_C140S was introduced into *E. coli* BL21 (DE3) cells to produce the inactive mutant recombinant protein as described previously.” (Page 29, line 651)

- In Results

“Moreover, expression of the inactive mutant GFP-EhPTEN1_C140S in *E. histolytica* trophozoites did not cause significant changes in pinocytosis, trogocytosis, and phagocytosis, which indicates that the enzymatic activity of EhPTEN1 is responsible for its phenotypes (S9 Fig).” (Page 13, line 297)

- In Figure legends

**“S9 Fig. Expression, localization, and phenotypic analysis of GFP-EhPTEN1_C140S. (A)** Schematic representation of EhPTEN1 and EhPTEN1_C140S mutant. Amino acid residues mutated are shown in red. **(B) I**mmunoblot of GFP-EhPTEN1_C140S and GFP (control) in *E. histolytica* transformants. Approximately 30 µg of total lysates from GFP mock-transfected control and GFP-EhPTEN1_C140S expressing transformant were subjected to SDS-PAGE and immunoblot analysis using anti-GFP antibody and anti-CS1 antibody. An arrow indicates GFP-EhPTEN1_C140S. **(C)** Localization of GFP-EhPTEN1_C140S in a quiescent state. (Scale bar, 10 μm). **(D)** The effect of GFP-EhPTEN1_C140S expression on pinocytosis. Trophozoites of GFP mock transfected and GFP-EhPTEN1_C140S expressing strains were mixed with RITC dextran and uptake was monitored as described in Materials and methods. Experiments were conducted twice independently and error bars indicating standard errors of two biological replicates. (E-F) Effect of GFP-EhPTEN1_C140S expression on trogocytosis (E) and phagocytosis (F). Trophozoites of GFP mock transfected and GFP-EhPTEN1_C140S expressing strains were incubated with live CHO cells **(E)** or heat killed CHO cells **(F)** that have been stained with CellTracker Orange to evaluate trogocytosis and phagocytosis, respectively. The images were captured on CQ1 as described in Materials and methods and analyzed to calculate the numbers of CHO cell-containing trogosomes or phagosomes per amoeba. Experiments were conducted twice independently in duplicates and error bars indicating standard errors of two biological replicates." (Page 38, line 855-872)

- A primer set used for construction of EhPTEN1_C140S were also added in S2 Table.

**Comment #14 (Reviewer #2 Comment #3):**

It is not clear what are the downstream pathways finally regulate endocytic processes. Questions such as the mechanism of recruitment of PTEN in pseudopods should be addressed.

**Our response:** We appreciate this valuable suggestion. The effect of EhPTEN1 on the downstream pathways in *E. histolytica* is not yet clear and will be addressed in the future study. However, we summarized the possible mechanism where EhPTEN1 regulates endocytic processes and motility in S12 Fig.

**Comment #15 (Reviewer #2 Comment #4):**

There should be an inactive mutant control for the enzyme assays (Table 1).

**Our response:** Thank you for the suggestion. According to the suggestion, we have produced recombinant inactive EhPTEN1 mutant and confirmed lack of activity towards PtdIns(3,4,5)P_3._ We also added a new supplementary figure (S10 Fig) for the conformation of EhPTEN1 inactive mutant expression and modified the text as follow:

- In Results: “Furthermore, we produced a mutant form of EhPTEN1, in which the cysteine residue implicated for catalysis is replaced with serine by site-directed mutagenesis (EhPTEN1_C140S) (S10 Fig). Recombinant EhPTEN1_C140S showed markedly lower phosphatase activity towards PtdIns(3,4,5)P_3_ when compared to wild type, with the low specific activity (0.77 ± 0.14 nmol/min/mg).” (Page 13, line 293)
- In the figure legend: “**S10 Fig. Production and activity of EhPTEN1_C140S.** **(A)** Expression and purification of recombinant EhPTEN1_C140S. Protein samples at each step of purification were subjected to 10% SDS-PAGE and the gel was stained with Coomassie Brilliant Blue. **(B)** Immunoblot analysis of purified recombinant EhPTEN1_C140S using anti-His-tag antibody. **(C)** Specific activity of recombinant EhPTEN1 and EhPTEN1_C140S using 100 μM of PtdIns(3,4,5)P_3_ as substrate. Reactions were carried out in a volume of 25 µl for 40 min at 37°C,and then terminated by the addition of 100 µl of malachite green reagent as described in the materials and methods. The absorbance at 630 nm was measured and phosphate released was quantified by comparison to a standard curve of inorganic phosphate. The mean ± S.D. of three independent experiments is shown.” (Page 39, line 873)

**Comment #16 (Reviewer #2):**

**Part III – Minor Issues: Editorial and Data Presentation Modifications**

For figure 1B, please explain more clearly in the figure legend what the assay is.

**Our response:** For clarification, we added an explanation in the figure legend of Fig 1B as follow:

“The steady-state mRNA levels of PTEN isotypes in *E. histolytica* were created using the data from our previous work [24-26] where transcriptome of trophozoites of *E. histolytica* HM-1:IMSS cl6 reference strain was analyzed using DNA microarrays technology [25]. The levels of steady-state mRNA of PTEN isotypes are shown after normalization against that of RNA polymerase II.”

**Comment #17 (Reviewer #2):**

The authors used “mock” to refer to control cells throughout the manuscript. More information is needed on what these mock cells are. Are they WT HM1:IMSS trophozoites or G3 trophozoites? Have they been transfected with the corresponding empty vectors or not?

**Our response:** We used a HM-1:IMSS-derived line that had been transformed with GFP-expressing empty plasmid (GFP) as a control for comparison with GFP-EhPTEN1, whereas a G3-derived line that had been transfected with the empty psAP2-Gunma vector (psAP2) was used for comparison against *EhPTEN1-*gene silenced strain in which *EhPTEN1* was gene silenced. We have added the description in the main text of the result section for clarification (page 8, line 165, page 10 lines 214 and 219, page 11 lines 234 and 241, and page 12 lines 253, 255, 260, and 262). We also corrected the labels and the figure legends (Fig 2, Fig 5-10, S5 Fig, S7 Fig).

**Comment #18 (Reviewer #2):**

The phosphatase activity of the recombinant protein is the highest for a different phospholipid than those phospholipids that are bound by the overexpressed protein from trophozoite lysates. Please comment on the differences and potential biological meaning of the differences.

**Our response:** We have added the comment regarding this issue on page 14, line 316:

“The binding of EhPTEN1 with diverse immobilized phospholipids may regulate its localization to different cellular compartment where EhPTEN1 exerts its phosphatase activity as has been shown previously that the binding of PtdIns(3)P with C2 domain of human PTEN target its localization to endosomal membranes [35]”

**Comment #19 (Reviewer #2):**

The argument that these studies could lead to the development of new control measures for Entamoeba histolytica seems a stretch. If the authors would like to make this argument, this would need to be strengthened with a stronger justification.

**Our response:** We have softened our statement as follow:

“Our findings help in the elucidation of the physiological significance of PTEN and cellular events regulated via phosphoinositides in this enteric parasite and other pathogenic parasites, and may potentially lead to the development of new control measures against parasitic diseases.” (Page 3, line 60)

We deleted the following statement: “Also, it may inform the rational design of novel therapies against eukaryotic pathogens.” (Page 19, the final statement in Discussion)

**Comment #20 (Reviewer #3):**

Fig. 1: The multiple alignment has been done with only three sequences that display fairly large diversity. It should be redone with a number of sequences from different species and all PTEN homologs should be included.

**Our response:** As suggested by the reviewer, we included PTEN sequences from other representative species in Fig 1C and modified the figure legend as follow:

**“**(C) Multiple amino acid sequence alignment of human PTEN (P60484), *Xenopus laevis* (AAD46165.1), *Drosophila melanogaster* (Q9Y0B6), *D. discoideum* PTEN (Q8T9S), and EhPTEN1 (XP_653141.2) was constructed by using clustalw algorithm (<http://clustalw.ddbj.nig.ac.jp)>.” (correction shown underlined)

We also added a new supplementary figure (S11 Fig) to include the sequence alignment of all PTEN isotypes from *E. histolytica.*

**Comment #21 (Reviewer #3):**

Discussion: It can be reduced and a working model to be included.

**Our response:** We have reduced Discussion and added a new supplementary figure to include the working model (S12 Fig).
